# Supplementary material for: Modeling glioblastoma heterogeneity as a dynamic network of cell states
Source: Mol Syst Biol. 2021 Sep 16;17(9):e10105. doi: 10.15252/msb.202010105 (PMC8444284; doi:10.15252/msb.202010105)
Supplement: Supplementary file 5 — Source Data for Figure 3 [file MSB-17-e10105-s001.zip › Figure3A_sourcedata/GSEA_3065/nosigdb_state4.GseaPreranked.1622718430994/RICHARDS_NOWAKOWSKI_SCIENCE_2017_NIN2_UPREG.html]

Details for gene set RICHARDS\_NOWAKOWSKI\_SCIENCE\_2017\_NIN2\_UPREG[GSEA]

|  || Dataset | state4 |
| Phenotype | NoPhenotypeAvailable |
| Upregulated in class | na\_pos |
| GeneSet | RICHARDS\_NOWAKOWSKI\_SCIENCE\_2017\_NIN2\_UPREG |
| Enrichment Score (ES) | 0.35672507 |
| Normalized Enrichment Score (NES) | 1.7953497 |
| Nominal p-value | 0.0 |
| FDR q-value | 0.008647121 |
| FWER p-Value | 0.078 |
Table: GSEA Results Summary

  

Fig 1: Enrichment plot: RICHARDS\_NOWAKOWSKI\_SCIENCE\_2017\_NIN2\_UPREG      
 Profile of the Running ES Score & Positions of GeneSet Members on the Rank Ordered List

  

| PROBE | GENE SYMBOL | GENE\_TITLE | RANK IN GENE LIST | RANK METRIC SCORE | RUNNING ES | CORE ENRICHMENT || 1 | FTL |  |  | 13 | 0.466 | 0.0091 | Yes |
| 2 | SOX4 |  |  | 18 | 0.402 | 0.0182 | Yes |
| 3 | TOMM7 |  |  | 22 | 0.380 | 0.0269 | Yes |
| 4 | MARCKS |  |  | 27 | 0.363 | 0.0350 | Yes |
| 5 | COMMD6 |  |  | 36 | 0.334 | 0.0418 | Yes |
| 6 | FEZ1 |  |  | 42 | 0.313 | 0.0485 | Yes |
| 7 | PFN2 |  |  | 46 | 0.303 | 0.0554 | Yes |
| 8 | ATP6V1G1 |  |  | 47 | 0.303 | 0.0627 | Yes |
| 9 | BNIP3L |  |  | 54 | 0.294 | 0.0688 | Yes |
| 10 | SEPT7 |  |  | 57 | 0.292 | 0.0756 | Yes |
| 11 | RPL34 |  |  | 69 | 0.278 | 0.0805 | Yes |
| 12 | CCNI |  |  | 76 | 0.266 | 0.0859 | Yes |
| 13 | RPS11 |  |  | 80 | 0.262 | 0.0918 | Yes |
| 14 | SMDT1 |  |  | 88 | 0.259 | 0.0969 | Yes |
| 15 | RPS27 |  |  | 98 | 0.250 | 0.1014 | Yes |
| 16 | SNRPN |  |  | 101 | 0.247 | 0.1070 | Yes |
| 17 | UBL5 |  |  | 106 | 0.245 | 0.1123 | Yes |
| 18 | MAP1B |  |  | 111 | 0.243 | 0.1175 | Yes |
| 19 | UQCRB |  |  | 114 | 0.241 | 0.1230 | Yes |
| 20 | PRDX5 |  |  | 123 | 0.237 | 0.1274 | Yes |
| 21 | RPL37A |  |  | 124 | 0.237 | 0.1332 | Yes |
| 22 | RPS28 |  |  | 141 | 0.226 | 0.1359 | Yes |
| 23 | RPL6 |  |  | 142 | 0.225 | 0.1414 | Yes |
| 24 | LAMTOR5 |  |  | 144 | 0.225 | 0.1467 | Yes |
| 25 | RPS27L |  |  | 149 | 0.223 | 0.1514 | Yes |
| 26 | COX7C |  |  | 162 | 0.216 | 0.1546 | Yes |
| 27 | RPS19 |  |  | 166 | 0.214 | 0.1593 | Yes |
| 28 | RPL39 |  |  | 168 | 0.214 | 0.1644 | Yes |
| 29 | RPS3A |  |  | 174 | 0.212 | 0.1686 | Yes |
| 30 | GPX4 |  |  | 183 | 0.210 | 0.1724 | Yes |
| 31 | COX6B1 |  |  | 185 | 0.209 | 0.1773 | Yes |
| 32 | RPS15A |  |  | 188 | 0.207 | 0.1820 | Yes |
| 33 | H3F3A |  |  | 189 | 0.207 | 0.1870 | Yes |
| 34 | BRK1 |  |  | 191 | 0.206 | 0.1919 | Yes |
| 35 | RPL9 |  |  | 196 | 0.205 | 0.1961 | Yes |
| 36 | RPL35A |  |  | 199 | 0.204 | 0.2008 | Yes |
| 37 | EID1 |  |  | 204 | 0.202 | 0.2050 | Yes |
| 38 | RPL21 |  |  | 213 | 0.198 | 0.2084 | Yes |
| 39 | TMA7 |  |  | 225 | 0.192 | 0.2112 | Yes |
| 40 | SMIM14 |  |  | 236 | 0.189 | 0.2141 | Yes |
| 41 | UBA52 |  |  | 239 | 0.188 | 0.2183 | Yes |
| 42 | RPL36 |  |  | 246 | 0.186 | 0.2218 | Yes |
| 43 | DCLK2 |  |  | 250 | 0.186 | 0.2258 | Yes |
| 44 | RPLP2 |  |  | 257 | 0.184 | 0.2293 | Yes |
| 45 | RPS20 |  |  | 259 | 0.184 | 0.2336 | Yes |
| 46 | COX6C |  |  | 269 | 0.181 | 0.2364 | Yes |
| 47 | RPS13 |  |  | 276 | 0.179 | 0.2398 | Yes |
| 48 | CAMTA1 |  |  | 282 | 0.178 | 0.2432 | Yes |
| 49 | TIMM8B |  |  | 283 | 0.178 | 0.2476 | Yes |
| 50 | RPL10 |  |  | 287 | 0.176 | 0.2513 | Yes |
| 51 | RPL7A |  |  | 294 | 0.174 | 0.2545 | Yes |
| 52 | DPYSL3 |  |  | 298 | 0.174 | 0.2582 | Yes |
| 53 | ZCRB1 |  |  | 302 | 0.173 | 0.2619 | Yes |
| 54 | RPL26 |  |  | 317 | 0.171 | 0.2637 | Yes |
| 55 | RPL38 |  |  | 327 | 0.170 | 0.2663 | Yes |
| 56 | C5orf24 |  |  | 355 | 0.163 | 0.2656 | Yes |
| 57 | TCF4 |  |  | 359 | 0.163 | 0.2691 | Yes |
| 58 | RPS16 |  |  | 366 | 0.161 | 0.2719 | Yes |
| 59 | RPS29 |  |  | 376 | 0.158 | 0.2742 | Yes |
| 60 | C19orf53 |  |  | 385 | 0.157 | 0.2767 | Yes |
| 61 | TERF2IP |  |  | 386 | 0.157 | 0.2805 | Yes |
| 62 | TBCA |  |  | 391 | 0.155 | 0.2836 | Yes |
| 63 | RPS27A |  |  | 395 | 0.153 | 0.2868 | Yes |
| 64 | POLR2J |  |  | 401 | 0.151 | 0.2896 | Yes |
| 65 | CRMP1 |  |  | 405 | 0.151 | 0.2927 | Yes |
| 66 | NEDD8 |  |  | 413 | 0.150 | 0.2952 | Yes |
| 67 | RAB14 |  |  | 425 | 0.147 | 0.2969 | Yes |
| 68 | ABAT |  |  | 435 | 0.145 | 0.2988 | Yes |
| 69 | CTDSP2 |  |  | 444 | 0.144 | 0.3010 | Yes |
| 70 | ANKIB1 |  |  | 459 | 0.143 | 0.3020 | Yes |
| 71 | KMT2E |  |  | 472 | 0.141 | 0.3034 | Yes |
| 72 | RPL22 |  |  | 473 | 0.141 | 0.3068 | Yes |
| 73 | MAP3K13 |  |  | 475 | 0.141 | 0.3101 | Yes |
| 74 | RPL24 |  |  | 515 | 0.134 | 0.3067 | Yes |
| 75 | NDUFA5 |  |  | 516 | 0.134 | 0.3099 | Yes |
| 76 | FAU |  |  | 518 | 0.134 | 0.3130 | Yes |
| 77 | ZNF106 |  |  | 528 | 0.133 | 0.3147 | Yes |
| 78 | RPL11 |  |  | 534 | 0.133 | 0.3171 | Yes |
| 79 | CCDC90B |  |  | 540 | 0.132 | 0.3194 | Yes |
| 80 | CHMP5 |  |  | 547 | 0.132 | 0.3216 | Yes |
| 81 | RPL18 |  |  | 549 | 0.131 | 0.3246 | Yes |
| 82 | RPL18A |  |  | 550 | 0.131 | 0.3278 | Yes |
| 83 | CRIPT |  |  | 556 | 0.131 | 0.3301 | Yes |
| 84 | THYN1 |  |  | 560 | 0.130 | 0.3327 | Yes |
| 85 | CALM3 |  |  | 570 | 0.129 | 0.3343 | Yes |
| 86 | ZFAS1 |  |  | 574 | 0.129 | 0.3369 | Yes |
| 87 | GOLGA7 |  |  | 586 | 0.127 | 0.3381 | Yes |
| 88 | TMSB10 |  |  | 616 | 0.124 | 0.3362 | Yes |
| 89 | KIF21A |  |  | 627 | 0.123 | 0.3374 | Yes |
| 90 | CAMLG |  |  | 639 | 0.121 | 0.3385 | Yes |
| 91 | DAZAP2 |  |  | 641 | 0.121 | 0.3413 | Yes |
| 92 | RPS25 |  |  | 642 | 0.121 | 0.3442 | Yes |
| 93 | TMEM263 |  |  | 654 | 0.120 | 0.3452 | Yes |
| 94 | ARL3 |  |  | 657 | 0.119 | 0.3478 | Yes |
| 95 | NDUFS5 |  |  | 681 | 0.117 | 0.3467 | Yes |
| 96 | COMMD3 |  |  | 694 | 0.115 | 0.3474 | Yes |
| 97 | RAB18 |  |  | 699 | 0.114 | 0.3495 | Yes |
| 98 | TCEAL7 |  |  | 705 | 0.113 | 0.3514 | Yes |
| 99 | KLHL24 |  |  | 726 | 0.111 | 0.3506 | Yes |
| 100 | RPS4Y1 |  |  | 733 | 0.110 | 0.3523 | Yes |
| 101 | EVL |  |  | 746 | 0.109 | 0.3528 | Yes |
| 102 | RPL27 |  |  | 749 | 0.109 | 0.3551 | Yes |
| 103 | MEG3 |  |  | 756 | 0.108 | 0.3567 | Yes |
| 104 | RAP2A |  |  | 781 | 0.105 | 0.3552 | No |
| 105 | PDCD4 |  |  | 796 | 0.104 | 0.3553 | No |
| 106 | SH3BGRL3 |  |  | 804 | 0.103 | 0.3566 | No |
| 107 | STAT2 |  |  | 824 | 0.102 | 0.3558 | No |
| 108 | PARK7 |  |  | 873 | 0.098 | 0.3500 | No |
| 109 | NDUFB3 |  |  | 883 | 0.097 | 0.3508 | No |
| 110 | TMEM14A |  |  | 899 | 0.096 | 0.3505 | No |
| 111 | SLAIN1 |  |  | 920 | 0.094 | 0.3494 | No |
| 112 | DYNC1LI2 |  |  | 939 | 0.093 | 0.3485 | No |
| 113 | LAMTOR3 |  |  | 943 | 0.092 | 0.3503 | No |
| 114 | NDUFA1 |  |  | 955 | 0.092 | 0.3506 | No |
| 115 | RFX3 |  |  | 970 | 0.090 | 0.3504 | No |
| 116 | SOX2 |  |  | 975 | 0.090 | 0.3519 | No |
| 117 | NDUFA4 |  |  | 977 | 0.090 | 0.3539 | No |
| 118 | GNAQ |  |  | 997 | 0.088 | 0.3528 | No |
| 119 | YWHAG |  |  | 1016 | 0.087 | 0.3518 | No |
| 120 | PNMA1 |  |  | 1054 | 0.084 | 0.3475 | No |
| 121 | ANKRD12 |  |  | 1072 | 0.083 | 0.3466 | No |
| 122 | PRKAR2B |  |  | 1084 | 0.083 | 0.3467 | No |
| 123 | MOB1B |  |  | 1091 | 0.083 | 0.3477 | No |
| 124 | EIF3E |  |  | 1124 | 0.080 | 0.3442 | No |
| 125 | NDUFS4 |  |  | 1144 | 0.079 | 0.3428 | No |
| 126 | GRSF1 |  |  | 1146 | 0.079 | 0.3446 | No |
| 127 | RBP1 |  |  | 1171 | 0.078 | 0.3424 | No |
| 128 | SMAD5 |  |  | 1186 | 0.077 | 0.3418 | No |
| 129 | RPL35 |  |  | 1187 | 0.077 | 0.3437 | No |
| 130 | ZSWIM6 |  |  | 1192 | 0.076 | 0.3449 | No |
| 131 | RNF38 |  |  | 1202 | 0.076 | 0.3452 | No |
| 132 | ACTR2 |  |  | 1224 | 0.075 | 0.3434 | No |
| 133 | ARHGAP5 |  |  | 1259 | 0.073 | 0.3393 | No |
| 134 | PSME1 |  |  | 1273 | 0.072 | 0.3388 | No |
| 135 | CCDC88A |  |  | 1279 | 0.072 | 0.3397 | No |
| 136 | RPL7 |  |  | 1306 | 0.071 | 0.3370 | No |
| 137 | KIFAP3 |  |  | 1310 | 0.070 | 0.3382 | No |
| 138 | FEM1B |  |  | 1316 | 0.070 | 0.3390 | No |
| 139 | APPBP2 |  |  | 1344 | 0.069 | 0.3361 | No |
| 140 | WDR26 |  |  | 1361 | 0.068 | 0.3350 | No |
| 141 | TRIP11 |  |  | 1375 | 0.067 | 0.3344 | No |
| 142 | NOVA2 |  |  | 1376 | 0.067 | 0.3360 | No |
| 143 | SCYL2 |  |  | 1384 | 0.067 | 0.3364 | No |
| 144 | PDCL |  |  | 1404 | 0.066 | 0.3348 | No |
| 145 | MIDN |  |  | 1414 | 0.066 | 0.3348 | No |
| 146 | SPAG9 |  |  | 1420 | 0.065 | 0.3355 | No |
| 147 | FZD3 |  |  | 1429 | 0.065 | 0.3358 | No |
| 148 | USP9X |  |  | 1438 | 0.065 | 0.3360 | No |
| 149 | SAP30BP |  |  | 1441 | 0.065 | 0.3372 | No |
| 150 | ATP6V1A |  |  | 1449 | 0.064 | 0.3375 | No |
| 151 | SLC30A9 |  |  | 1474 | 0.063 | 0.3349 | No |
| 152 | CNOT2 |  |  | 1479 | 0.063 | 0.3358 | No |
| 153 | CASC4 |  |  | 1487 | 0.062 | 0.3361 | No |
| 154 | ZBTB10 |  |  | 1490 | 0.062 | 0.3373 | No |
| 155 | CAMSAP2 |  |  | 1497 | 0.062 | 0.3377 | No |
| 156 | UFSP2 |  |  | 1499 | 0.062 | 0.3391 | No |
| 157 | CREBRF |  |  | 1511 | 0.061 | 0.3387 | No |
| 158 | COPS2 |  |  | 1548 | 0.060 | 0.3340 | No |
| 159 | EP300 |  |  | 1556 | 0.060 | 0.3342 | No |
| 160 | AMOTL2 |  |  | 1616 | 0.058 | 0.3255 | No |
| 161 | KRAS |  |  | 1657 | 0.057 | 0.3200 | No |
| 162 | INTS10 |  |  | 1662 | 0.057 | 0.3207 | No |
| 163 | DCTN4 |  |  | 1702 | 0.055 | 0.3153 | No |
| 164 | DDX1 |  |  | 1705 | 0.055 | 0.3163 | No |
| 165 | PCNP |  |  | 1750 | 0.054 | 0.3101 | No |
| 166 | PGD |  |  | 1767 | 0.053 | 0.3086 | No |
| 167 | IBTK |  |  | 1783 | 0.053 | 0.3073 | No |
| 168 | RPS21 |  |  | 1796 | 0.052 | 0.3065 | No |
| 169 | CMIP |  |  | 1800 | 0.052 | 0.3073 | No |
| 170 | RABGAP1 |  |  | 1814 | 0.052 | 0.3063 | No |
| 171 | ZC2HC1A |  |  | 1832 | 0.051 | 0.3047 | No |
| 172 | ARIH1 |  |  | 1857 | 0.051 | 0.3018 | No |
| 173 | SENP6 |  |  | 1861 | 0.050 | 0.3025 | No |
| 174 | MIB1 |  |  | 1865 | 0.050 | 0.3032 | No |
| 175 | MPC1 |  |  | 1881 | 0.050 | 0.3018 | No |
| 176 | AP3S1 |  |  | 1884 | 0.050 | 0.3027 | No |
| 177 | FAM177A1 |  |  | 1900 | 0.049 | 0.3013 | No |
| 178 | STARD3NL |  |  | 1916 | 0.049 | 0.2999 | No |
| 179 | PALLD |  |  | 1930 | 0.049 | 0.2989 | No |
| 180 | ZNF260 |  |  | 1934 | 0.049 | 0.2995 | No |
| 181 | AGO1 |  |  | 1936 | 0.049 | 0.3006 | No |
| 182 | KAT6B |  |  | 1983 | 0.047 | 0.2938 | No |
| 183 | MTSS1 |  |  | 1988 | 0.047 | 0.2943 | No |
| 184 | AKAP9 |  |  | 2002 | 0.047 | 0.2932 | No |
| 185 | ZYG11B |  |  | 2009 | 0.047 | 0.2933 | No |
| 186 | ZEB2 |  |  | 2031 | 0.046 | 0.2908 | No |
| 187 | EAF1 |  |  | 2032 | 0.046 | 0.2919 | No |
| 188 | PHAX |  |  | 2050 | 0.045 | 0.2901 | No |
| 189 | MINOS1 |  |  | 2071 | 0.045 | 0.2878 | No |
| 190 | ANKRD17 |  |  | 2095 | 0.044 | 0.2849 | No |
| 191 | CBX1 |  |  | 2104 | 0.044 | 0.2846 | No |
| 192 | MZT2B |  |  | 2107 | 0.044 | 0.2853 | No |
| 193 | PTPRA |  |  | 2113 | 0.043 | 0.2855 | No |
| 194 | FKBP3 |  |  | 2130 | 0.043 | 0.2838 | No |
| 195 | MED4 |  |  | 2138 | 0.043 | 0.2836 | No |
| 196 | LARP7 |  |  | 2188 | 0.042 | 0.2762 | No |
| 197 | YAF2 |  |  | 2206 | 0.041 | 0.2743 | No |
| 198 | TBC1D23 |  |  | 2208 | 0.041 | 0.2752 | No |
| 199 | SYNJ2BP |  |  | 2211 | 0.041 | 0.2758 | No |
| 200 | ACADM |  |  | 2224 | 0.041 | 0.2748 | No |
| 201 | MPRIP |  |  | 2277 | 0.040 | 0.2668 | No |
| 202 | TBRG1 |  |  | 2298 | 0.039 | 0.2643 | No |
| 203 | KAT7 |  |  | 2324 | 0.038 | 0.2610 | No |
| 204 | VPS35 |  |  | 2364 | 0.038 | 0.2552 | No |
| 205 | CNOT8 |  |  | 2383 | 0.037 | 0.2530 | No |
| 206 | DAP3 |  |  | 2419 | 0.037 | 0.2479 | No |
| 207 | BMPR2 |  |  | 2440 | 0.036 | 0.2454 | No |
| 208 | AGL |  |  | 2452 | 0.036 | 0.2443 | No |
| 209 | KARS |  |  | 2463 | 0.036 | 0.2435 | No |
| 210 | XPO7 |  |  | 2465 | 0.036 | 0.2442 | No |
| 211 | USP42 |  |  | 2470 | 0.035 | 0.2444 | No |
| 212 | PAPOLA |  |  | 2493 | 0.035 | 0.2414 | No |
| 213 | CLTC |  |  | 2535 | 0.034 | 0.2352 | No |
| 214 | APPL1 |  |  | 2556 | 0.034 | 0.2326 | No |
| 215 | HELZ |  |  | 2567 | 0.033 | 0.2317 | No |
| 216 | CRBN |  |  | 2578 | 0.033 | 0.2308 | No |
| 217 | TMEM170A |  |  | 2589 | 0.033 | 0.2299 | No |
| 218 | EXOC5 |  |  | 2593 | 0.033 | 0.2302 | No |
| 219 | RAB30 |  |  | 2595 | 0.033 | 0.2308 | No |
| 220 | ARL14EP |  |  | 2651 | 0.032 | 0.2221 | No |
| 221 | ROCK2 |  |  | 2662 | 0.031 | 0.2212 | No |
| 222 | ZNF644 |  |  | 2671 | 0.031 | 0.2206 | No |
| 223 | PICALM |  |  | 2677 | 0.031 | 0.2205 | No |
| 224 | ARRDC3 |  |  | 2741 | 0.030 | 0.2104 | No |
| 225 | PPFIA1 |  |  | 2765 | 0.029 | 0.2072 | No |
| 226 | UBN2 |  |  | 2767 | 0.029 | 0.2077 | No |
| 227 | SRGAP2B |  |  | 2770 | 0.029 | 0.2081 | No |
| 228 | KIAA0355 |  |  | 2797 | 0.029 | 0.2043 | No |
| 229 | GRIA4 |  |  | 2813 | 0.028 | 0.2024 | No |
| 230 | CNBP |  |  | 2838 | 0.028 | 0.1990 | No |
| 231 | CNOT7 |  |  | 2841 | 0.028 | 0.1993 | No |
| 232 | YY1 |  |  | 2849 | 0.027 | 0.1987 | No |
| 233 | GCC2 |  |  | 2871 | 0.027 | 0.1958 | No |
| 234 | PPP2R5C |  |  | 2887 | 0.027 | 0.1939 | No |
| 235 | TANK |  |  | 2890 | 0.027 | 0.1942 | No |
| 236 | RSBN1 |  |  | 2922 | 0.026 | 0.1895 | No |
| 237 | RAD23B |  |  | 2951 | 0.026 | 0.1853 | No |
| 238 | TRIP12 |  |  | 3003 | 0.025 | 0.1772 | No |
| 239 | KIAA2026 |  |  | 3041 | 0.024 | 0.1714 | No |
| 240 | PJA1 |  |  | 3047 | 0.024 | 0.1711 | No |
| 241 | STX16 |  |  | 3067 | 0.024 | 0.1685 | No |
| 242 | PELI1 |  |  | 3130 | 0.022 | 0.1584 | No |
| 243 | MPHOSPH8 |  |  | 3139 | 0.022 | 0.1575 | No |
| 244 | BICD2 |  |  | 3164 | 0.022 | 0.1539 | No |
| 245 | SMARCC2 |  |  | 3176 | 0.022 | 0.1526 | No |
| 246 | GID8 |  |  | 3187 | 0.021 | 0.1514 | No |
| 247 | PPP2R3A |  |  | 3199 | 0.021 | 0.1500 | No |
| 248 | DNAJC8 |  |  | 3237 | 0.020 | 0.1441 | No |
| 249 | MSI2 |  |  | 3242 | 0.020 | 0.1439 | No |
| 250 | NUFIP2 |  |  | 3258 | 0.020 | 0.1418 | No |
| 251 | ARFGEF1 |  |  | 3273 | 0.019 | 0.1399 | No |
| 252 | COPS6 |  |  | 3280 | 0.019 | 0.1393 | No |
| 253 | C11orf58 |  |  | 3282 | 0.019 | 0.1396 | No |
| 254 | QRICH1 |  |  | 3300 | 0.018 | 0.1371 | No |
| 255 | ZNF91 |  |  | 3311 | 0.018 | 0.1359 | No |
| 256 | TSC22D2 |  |  | 3343 | 0.017 | 0.1310 | No |
| 257 | HDAC2 |  |  | 3407 | 0.015 | 0.1205 | No |
| 258 | NIPBL |  |  | 3414 | 0.015 | 0.1199 | No |
| 259 | MAGEH1 |  |  | 3417 | 0.015 | 0.1199 | No |
| 260 | VEZF1 |  |  | 3424 | 0.015 | 0.1192 | No |
| 261 | CELF1 |  |  | 3441 | 0.014 | 0.1168 | No |
| 262 | MAGI2 |  |  | 3482 | 0.013 | 0.1103 | No |
| 263 | ZBTB44 |  |  | 3501 | 0.012 | 0.1075 | No |
| 264 | SBF2 |  |  | 3504 | 0.012 | 0.1074 | No |
| 265 | SMARCA5 |  |  | 3518 | 0.011 | 0.1055 | No |
| 266 | CHD6 |  |  | 3530 | 0.011 | 0.1038 | No |
| 267 | MSL1 |  |  | 3533 | 0.011 | 0.1038 | No |
| 268 | CNOT4 |  |  | 3536 | 0.011 | 0.1037 | No |
| 269 | COPB2 |  |  | 3552 | 0.011 | 0.1014 | No |
| 270 | PFDN1 |  |  | 3571 | 0.010 | 0.0985 | No |
| 271 | USP8 |  |  | 3574 | 0.010 | 0.0984 | No |
| 272 | ARF1 |  |  | 3581 | 0.009 | 0.0976 | No |
| 273 | CLIP1 |  |  | 3585 | 0.009 | 0.0973 | No |
| 274 | PPM1L |  |  | 3606 | 0.009 | 0.0941 | No |
| 275 | ZC3H7A |  |  | 3607 | 0.009 | 0.0943 | No |
| 276 | CEBPZOS |  |  | 3618 | 0.008 | 0.0928 | No |
| 277 | BROX |  |  | 3623 | 0.008 | 0.0923 | No |
| 278 | GOLGA4 |  |  | 3644 | 0.007 | 0.0891 | No |
| 279 | LEPROT |  |  | 3646 | 0.007 | 0.0891 | No |
| 280 | MAP4 |  |  | 3672 | 0.006 | 0.0849 | No |
| 281 | DENND4C |  |  | 3678 | 0.006 | 0.0842 | No |
| 282 | PPP1R10 |  |  | 3684 | 0.006 | 0.0835 | No |
| 283 | PPP2R2A |  |  | 3686 | 0.006 | 0.0835 | No |
| 284 | MED28 |  |  | 3698 | 0.005 | 0.0817 | No |
| 285 | NCOR1 |  |  | 3709 | 0.005 | 0.0801 | No |
| 286 | ACAP2 |  |  | 3715 | 0.005 | 0.0794 | No |
| 287 | PCMTD1 |  |  | 3716 | 0.004 | 0.0795 | No |
| 288 | DHX30 |  |  | 3719 | 0.004 | 0.0792 | No |
| 289 | DICER1 |  |  | 3720 | 0.004 | 0.0793 | No |
| 290 | ACAT1 |  |  | 3723 | 0.004 | 0.0791 | No |
| 291 | HECTD4 |  |  | 3727 | 0.004 | 0.0787 | No |
| 292 | CSNK1E |  |  | 3743 | 0.004 | 0.0762 | No |
| 293 | PPP1R12A |  |  | 3762 | 0.002 | 0.0732 | No |
| 294 | LSAMP |  |  | 3779 | 0.001 | 0.0705 | No |
| 295 | CLASP2 |  |  | 3780 | 0.001 | 0.0705 | No |
| 296 | RAB3GAP2 |  |  | 3789 | 0.001 | 0.0691 | No |
| 297 | CADM1 |  |  | 3798 | 0.001 | 0.0678 | No |
| 298 | PWP1 |  |  | 3853 | -0.002 | 0.0586 | No |
| 299 | MAP4K5 |  |  | 3873 | -0.003 | 0.0554 | No |
| 300 | MED10 |  |  | 3876 | -0.003 | 0.0551 | No |
| 301 | GAPVD1 |  |  | 3895 | -0.003 | 0.0521 | No |
| 302 | KAT6A |  |  | 3925 | -0.005 | 0.0472 | No |
| 303 | STAT3 |  |  | 3937 | -0.006 | 0.0455 | No |
| 304 | ROCK1 |  |  | 3943 | -0.006 | 0.0448 | No |
| 305 | OGT |  |  | 3974 | -0.008 | 0.0398 | No |
| 306 | PPP1R2 |  |  | 4009 | -0.010 | 0.0342 | No |
| 307 | AQR |  |  | 4017 | -0.010 | 0.0333 | No |
| 308 | HIP1 |  |  | 4043 | -0.012 | 0.0293 | No |
| 309 | C3orf14 |  |  | 4079 | -0.014 | 0.0236 | No |
| 310 | NDUFB8 |  |  | 4100 | -0.015 | 0.0206 | No |
| 311 | RALGAPA1 |  |  | 4104 | -0.016 | 0.0204 | No |
| 312 | ZC3H13 |  |  | 4154 | -0.019 | 0.0125 | No |
| 313 | KIAA1549 |  |  | 4163 | -0.020 | 0.0116 | No |
| 314 | GPATCH2L |  |  | 4168 | -0.020 | 0.0114 | No |
| 315 | GABBR1 |  |  | 4170 | -0.020 | 0.0117 | No |
| 316 | DNM3 |  |  | 4177 | -0.020 | 0.0112 | No |
| 317 | BIRC6 |  |  | 4222 | -0.023 | 0.0042 | No |
| 318 | TRIM13 |  |  | 4224 | -0.024 | 0.0046 | No |
| 319 | PSMC4 |  |  | 4249 | -0.025 | 0.0011 | No |
| 320 | EIF2AK2 |  |  | 4256 | -0.025 | 0.0007 | No |
| 321 | TNPO3 |  |  | 4283 | -0.027 | -0.0031 | No |
| 322 | HUWE1 |  |  | 4296 | -0.028 | -0.0045 | No |
| 323 | HLTF |  |  | 4299 | -0.029 | -0.0042 | No |
| 324 | KIDINS220 |  |  | 4314 | -0.030 | -0.0058 | No |
| 325 | YIPF5 |  |  | 4320 | -0.031 | -0.0059 | No |
| 326 | PHF20 |  |  | 4333 | -0.031 | -0.0072 | No |
| 327 | EPC1 |  |  | 4348 | -0.032 | -0.0089 | No |
| 328 | PHF20L1 |  |  | 4359 | -0.033 | -0.0098 | No |
| 329 | ZNF506 |  |  | 4372 | -0.034 | -0.0110 | No |
| 330 | REEP3 |  |  | 4373 | -0.034 | -0.0102 | No |
| 331 | WDR60 |  |  | 4380 | -0.034 | -0.0104 | No |
| 332 | BRAF |  |  | 4397 | -0.036 | -0.0123 | No |
| 333 | SRSF11 |  |  | 4400 | -0.036 | -0.0118 | No |
| 334 | PLK2 |  |  | 4403 | -0.036 | -0.0112 | No |
| 335 | SCAF11 |  |  | 4415 | -0.037 | -0.0122 | No |
| 336 | H2AFY |  |  | 4419 | -0.037 | -0.0119 | No |
| 337 | OFD1 |  |  | 4441 | -0.039 | -0.0145 | No |
| 338 | MRPL42 |  |  | 4445 | -0.040 | -0.0141 | No |
| 339 | ZNF721 |  |  | 4449 | -0.040 | -0.0136 | No |
| 340 | RNMT |  |  | 4452 | -0.040 | -0.0130 | No |
| 341 | TMEM98 |  |  | 4455 | -0.041 | -0.0123 | No |
| 342 | UBR3 |  |  | 4462 | -0.041 | -0.0124 | No |
| 343 | TRIM24 |  |  | 4466 | -0.042 | -0.0119 | No |
| 344 | MUM1 |  |  | 4480 | -0.043 | -0.0130 | No |
| 345 | SNRPF |  |  | 4498 | -0.044 | -0.0149 | No |
| 346 | SCARB2 |  |  | 4507 | -0.045 | -0.0152 | No |
| 347 | CASK |  |  | 4533 | -0.047 | -0.0183 | No |
| 348 | PPHLN1 |  |  | 4553 | -0.048 | -0.0204 | No |
| 349 | SYT11 |  |  | 4599 | -0.051 | -0.0269 | No |
| 350 | PTCD3 |  |  | 4607 | -0.052 | -0.0268 | No |
| 351 | PRPF40A |  |  | 4617 | -0.052 | -0.0271 | No |
| 352 | ZNF638 |  |  | 4625 | -0.053 | -0.0270 | No |
| 353 | HERC2 |  |  | 4631 | -0.054 | -0.0266 | No |
| 354 | ITGB1BP1 |  |  | 4660 | -0.056 | -0.0300 | No |
| 355 | BOD1L1 |  |  | 4666 | -0.057 | -0.0295 | No |
| 356 | OLA1 |  |  | 4718 | -0.061 | -0.0368 | No |
| 357 | SETD5 |  |  | 4788 | -0.067 | -0.0470 | No |
| 358 | SON |  |  | 4803 | -0.068 | -0.0477 | No |
| 359 | THOC2 |  |  | 4814 | -0.069 | -0.0478 | No |
| 360 | DHX29 |  |  | 4840 | -0.071 | -0.0503 | No |
| 361 | MRPL47 |  |  | 4847 | -0.072 | -0.0496 | No |
| 362 | MRPS5 |  |  | 4848 | -0.072 | -0.0479 | No |
| 363 | CDK13 |  |  | 4859 | -0.072 | -0.0478 | No |
| 364 | ZNF704 |  |  | 4861 | -0.072 | -0.0462 | No |
| 365 | PAXBP1 |  |  | 4878 | -0.074 | -0.0472 | No |
| 366 | TXN |  |  | 4891 | -0.074 | -0.0474 | No |
| 367 | TOMM20 |  |  | 4901 | -0.075 | -0.0472 | No |
| 368 | TMEM87A |  |  | 4932 | -0.077 | -0.0504 | No |
| 369 | PRRC2C |  |  | 4940 | -0.078 | -0.0497 | No |
| 370 | ZNF207 |  |  | 4944 | -0.079 | -0.0483 | No |
| 371 | EIF5 |  |  | 4991 | -0.082 | -0.0542 | No |
| 372 | HCG18 |  |  | 5005 | -0.083 | -0.0545 | No |
| 373 | PPA1 |  |  | 5017 | -0.084 | -0.0543 | No |
| 374 | TMSB4X |  |  | 5045 | -0.087 | -0.0568 | No |
| 375 | TOP1 |  |  | 5082 | -0.090 | -0.0608 | No |
| 376 | NDUFA9 |  |  | 5088 | -0.091 | -0.0595 | No |
| 377 | IPO9 |  |  | 5093 | -0.091 | -0.0580 | No |
| 378 | TPRKB |  |  | 5099 | -0.092 | -0.0566 | No |
| 379 | RSBN1L |  |  | 5123 | -0.093 | -0.0583 | No |
| 380 | PSMD6 |  |  | 5160 | -0.098 | -0.0620 | No |
| 381 | SNRNP40 |  |  | 5174 | -0.099 | -0.0619 | No |
| 382 | ATP11B |  |  | 5175 | -0.099 | -0.0595 | No |
| 383 | TPR |  |  | 5222 | -0.103 | -0.0649 | No |
| 384 | HMGN1 |  |  | 5223 | -0.103 | -0.0624 | No |
| 385 | TMEM123 |  |  | 5280 | -0.108 | -0.0693 | No |
| 386 | DHX9 |  |  | 5302 | -0.110 | -0.0703 | No |
| 387 | KMT2A |  |  | 5358 | -0.114 | -0.0769 | No |
| 388 | CNTRL |  |  | 5363 | -0.115 | -0.0748 | No |
| 389 | CBX3 |  |  | 5377 | -0.116 | -0.0742 | No |
| 390 | KLHL23 |  |  | 5397 | -0.118 | -0.0746 | No |
| 391 | TRAM1 |  |  | 5439 | -0.123 | -0.0787 | No |
| 392 | FNBP4 |  |  | 5456 | -0.126 | -0.0784 | No |
| 393 | SUCO |  |  | 5462 | -0.126 | -0.0762 | No |
| 394 | ANKRD36C |  |  | 5516 | -0.133 | -0.0820 | No |
| 395 | CCDC82 |  |  | 5521 | -0.134 | -0.0795 | No |
| 396 | NT5C3A |  |  | 5540 | -0.135 | -0.0793 | No |
| 397 | MIA3 |  |  | 5545 | -0.136 | -0.0767 | No |
| 398 | ST8SIA5 |  |  | 5567 | -0.138 | -0.0769 | No |
| 399 | TMCO1 |  |  | 5576 | -0.140 | -0.0749 | No |
| 400 | SUN1 |  |  | 5604 | -0.145 | -0.0760 | No |
| 401 | SOX6 |  |  | 5612 | -0.146 | -0.0737 | No |
| 402 | NELFCD |  |  | 5614 | -0.146 | -0.0703 | No |
| 403 | NUP50 |  |  | 5619 | -0.147 | -0.0674 | No |
| 404 | TM9SF3 |  |  | 5651 | -0.152 | -0.0690 | No |
| 405 | U2SURP |  |  | 5659 | -0.153 | -0.0665 | No |
| 406 | ORMDL1 |  |  | 5661 | -0.153 | -0.0630 | No |
| 407 | RBM33 |  |  | 5680 | -0.157 | -0.0622 | No |
| 408 | MSH2 |  |  | 5711 | -0.162 | -0.0635 | No |
| 409 | CACYBP |  |  | 5713 | -0.162 | -0.0597 | No |
| 410 | DERL2 |  |  | 5762 | -0.175 | -0.0637 | No |
| 411 | RSRC1 |  |  | 5766 | -0.176 | -0.0599 | No |
| 412 | ZNF738 |  |  | 5768 | -0.177 | -0.0558 | No |
| 413 | MALAT1 |  |  | 5783 | -0.179 | -0.0539 | No |
| 414 | MSH6 |  |  | 5799 | -0.182 | -0.0520 | No |
| 415 | HAT1 |  |  | 5803 | -0.184 | -0.0480 | No |
| 416 | LSM8 |  |  | 5822 | -0.188 | -0.0466 | No |
| 417 | CD46 |  |  | 5846 | -0.194 | -0.0458 | No |
| 418 | PTMA |  |  | 5852 | -0.195 | -0.0419 | No |
| 419 | ERP44 |  |  | 5856 | -0.196 | -0.0377 | No |
| 420 | SPCS1 |  |  | 5857 | -0.196 | -0.0329 | No |
| 421 | PAICS |  |  | 5858 | -0.196 | -0.0281 | No |
| 422 | HIST1H1E |  |  | 5865 | -0.198 | -0.0244 | No |
| 423 | MTDH |  |  | 5882 | -0.202 | -0.0222 | No |
| 424 | SOX11 |  |  | 5885 | -0.203 | -0.0176 | No |
| 425 | BTG3 |  |  | 5895 | -0.206 | -0.0142 | No |
| 426 | SEC11A |  |  | 5933 | -0.216 | -0.0152 | No |
| 427 | SPCS3 |  |  | 5948 | -0.220 | -0.0123 | No |
| 428 | SRSF3 |  |  | 5969 | -0.227 | -0.0102 | No |
| 429 | MAGI1 |  |  | 5985 | -0.233 | -0.0071 | No |
| 430 | STMN1 |  |  | 5993 | -0.236 | -0.0026 | No |
| 431 | CREBZF |  |  | 6011 | -0.242 | 0.0004 | No |
| 432 | PHTF2 |  |  | 6018 | -0.245 | 0.0053 | No |
| 433 | HNRNPDL |  |  | 6028 | -0.251 | 0.0098 | No |
| 434 | LEPROTL1 |  |  | 6044 | -0.266 | 0.0137 | No |
| 435 | HNRNPH1 |  |  | 6068 | -0.277 | 0.0165 | No |
| 436 | LBR |  |  | 6118 | -0.312 | 0.0157 | No |
| 437 | FUS |  |  | 6170 | -0.371 | 0.0160 | No |
Table: GSEA details [plain text format]

  

Fig 2: RICHARDS\_NOWAKOWSKI\_SCIENCE\_2017\_NIN2\_UPREG: Random ES distribution      
 Gene set null distribution of ES for **RICHARDS\_NOWAKOWSKI\_SCIENCE\_2017\_NIN2\_UPREG**
